# Experimental Study on the Degradation Mechanism of BFRP Under the Coupling Effect of Chloride Freeze-Thaw Cycles

**DOI:** 10.3390/polym17192654

**Published:** 2025-09-30

**Authors:** Zhigang Gao, Tao He, Qing Qin, Chenghua Zhang, Zhe Wang, Qi Lin, Yuhao Hei

**Affiliations:** 1 School of Civil and Architecture Engineering, Xi’an University of Science and Technology, Xi’an 710054, China; gaozhigang@xust.edu.cn (Z.G.); qinqingjd@163.com (Q.Q.); zch-0819@163.com (C.Z.); wz12336@outlook.com (Z.W.); 2 School-Enterprise Joint Research, Center of Underground, Structure Eathquakem Resistance, Shaanxi Province “Four·Main Bodies·and·One·Joint”, Xi’an 710054, China; 3 Xi’an XD New Energy Co., Ltd., Xi’an 710075, China; linqi@xd.cee-group.cn (Q.L.); heiyuhao@xd.xee-group.cn (Y.H.)

**Keywords:** coupled chloride freeze-thaw environment, basalt fiber reinforced polymer, durability performance, microscopic mechanism

## Abstract

Basalt fiber reinforced polymer (BFRP) is one of the new materials that can be used for making photovoltaic scaffolds, which can effectively solve the problem of the rapid deterioration of complex environmental performance and high maintenance cost of traditional scaffold materials. This paper focuses on the BFRP photovoltaic support in the cold and arid irrigation area of northwest China, carries out the durability test under the action of chloride salt, freeze-thaw cycle, and chloride salt freeze-thaw environment coupling, and it compares and analyzes the degradation law of the mechanical properties of BFRP sheets under different environmental effects. The performance degradation mechanism of BFRP materials under different environmental effects was revealed by SEM scanning electron microscopy and EDS energy spectrum analysis. The main conclusions are as follows: (1) Under the action of chloride salt, the tensile strength, elastic modulus and elongation at break of the specimen decreased by 11.46%, 7.02%, and 10.27%, respectively. Under the freeze-thaw cycle, the tensile strength and elongation at break of the specimen decreased by 9.62% and 6.85%, while the elastic modulus first increased and then decreased, with a maximum decrease of 12.95%. The degradation of mechanical properties is the most serious under the coupling effect of chloride salt and the freeze-thaw environment. The tensile strength, elastic modulus, and elongation at break of the specimens decreased by 25.73%, 9.55%, and 24.81%, respectively. (2) In the chloride environment, the distribution of elements on the surface of the specimen changed, the metal ions of the fibers precipitated, and ‘black spots‘ and corrosion pits appeared. The resin matrix forms ‘sponge-like‘ pores; under the freeze-thaw cycle, the fiber–resin interface cracks and fiber shedding intensifies; under the coupling effect of chloride freeze-thaw, ‘black spots‘, pits, resin holes, and interface cracks increased, and chloride penetration corrosion accelerated.

## 1. Introduction

With the rapid expansion of photovoltaic projects, pressures on land resources are increasing, and new photovoltaic projects are extending to areas with complex terrain, as follows: in the northwest region of China, the cold and arid irrigation areas in this area are widely distributed, and the coupling effect of chloride erosion and freeze-thaw cycles is significant, which increases the performance requirements of engineering materials. The durability of traditional supports (galvanized steel and aluminum alloys) is easy to degrade under various complex extreme weather conditions in the northwest, and the maintenance cost is high, resulting in limited service life. It is urgent to replace traditional support materials with photovoltaic support materials that are lightweight and demonstrate strong extreme environmental adaptability [1,2,3,4].

Basalt fiber reinforced polymer, because of its light weight, high strength, strong corrosion resistance, convenient application and operation, and excellent damping performance, is one of the new materials that can be used for making photovoltaic stents, and it has received extensive attention from scholars at home and abroad. The creep behavior of BFRP prestressing tendons in salt solution was studied, and the creep degradation mechanism of BFRP prestressing tendons in salt solution was revealed [5]. The long-term durability degradation of the BFRP sheet and epoxy resin matrix in the dry–wet cycle environment containing chloride ions was studied, and the degradation mechanism was analyzed by SEM and the void volume fraction. The results show that the degradation of the material is mainly due to the destruction of the interface; salt precipitation accelerates the debonding of the interface, and the hydrolysis of the epoxy resin matrix produces many voids [6]. Durability tests of BFRP sheets under the dry–wet cycle conditions of sulfate, chloride, acidic, and alkaline solutions for up to 1 year were carried out. The results show that the performance degradation of BFRP is mainly reflected in the tensile strength, rather than the elastic modulus. Under the conditions of a dry–wet cycle, the performance degradation of BFRP in sulfate and chloride solution is more significant than that in acid and alkaline solution [7]. The durability of BFRP bars in an alkaline environment at different temperatures was studied, and the microscopic analysis was carried out by SEM and other methods. The results showed that resin hydrolysis, fiber–resin interface debonding, and fiber damage after corrosion were the main factors leading to the deterioration of BFRP bars [8]. The durability of BFRP sheets in deionized water and alkaline solution at 60 °C was studied. Combined with SEM analysis, the degradation mechanism of BFRP in deionized water and alkaline solution was discussed, and a new corrosion method was proposed to predict the long-term water absorption and tensile strength of BFRP [9]. The degradation of the fatigue performance of BFRP in salt solution was studied, and the damage evolution process was recorded by in situ SEM, which revealed the fatigue degradation performance of BFRP after corrosion in salt solution [10]. Considering the influence of BFRP-related parameters and temperature, the durability of BFRP bars in water, acid, salt, and alkali solutions was studied. The results show that the tensile strength of BFRP bars degrades faster in alkaline and water environments, followed by the acidic solution, and the greatest durability is demonstrated in salt solution [11]. The durability tests of BFRP bars in alkaline solution, salt solution, acidic solution, and deionized water solution at 25 °C, 40 °C, and 55 °C were carried out, and the degradation mechanism of BFRP bars was revealed by SEM. The results show that the influence of acid, salt, and deionized water on the durability of BFRP bars is less than that of alkaline solution [12]. With the support of a large number of experimental data studies, the effects of thermal aging, hygrothermal aging, seawater, acid–base environments, ultraviolet radiation, and stress cycle on BFRP were extensively discussed. At the same time, failure mechanisms such as delamination, fiber and matrix performance degradation, poor interfacial adhesion, and matrix surface cracking were discussed. These failure mechanisms are more common in FRP composites under hygrothermal and acidic environmental conditions [13]. It was shown that BFRP can produce inevitable water absorption in various environments, which will lead to expansion, plasticization, matrix hydrolysis, chemical changes, and fiber/matrix interface debonding, ultimately leading to the degradation of its mechanical properties [14]. The durability of phenolic basalt fiber reinforced polymer (P-BFRP) bars under high temperature was studied. The results showed that the mass of BFRP had a similar variation trend in air, but the degradation rate was slow in nitrogen. Below 350 °C, the specific heat capacity and thermal conductivity of all BFRP bars are similar, and the elastic modulus degradation trend is similar due to the influence of non-uniform stress distribution on fibers and fiber bundles [15]. The freeze-thaw durability of BFRP single-lap joints (SLJ) was experimentally and numerically studied. The results showed that the bonding properties of epoxy resin and fiber matrix interface were significantly degraded [16]. studied the durability of BFRP under dry–wet cycles in an alkaline environment and analyzed its mechanism by SEM. The deterioration mechanism of BFRP under dry–wet cycles and alkali was revealed, and the time shift factor for predicting the durability of BFRP bars under dry–wet cycles was proposed [17].

In summary, at present, basalt fiber composite materials research mainly focuses on durability under ultraviolet light, acid–base, chloride salt, and alternating dry–wet environments, and a few scholars have explored the degradation mechanism of BFRP under the action of freeze-thaw cycles. However, research on the coupling of chloride salt and freeze-thaw environments in the cold and arid irrigation area of northwest China is almost nonexistent.

In this paper, the research focuses on the cold and arid irrigation areas in the northwest. In order to solve the problem of durability degradation and service reliability of photovoltaic support under the coupling of chloride environments and freeze-thaw cycles, the following three groups of tests were carried out: 5% NaCl chloride environment corrosion from 0 to 150 days; the freeze-thaw cycle center temperature was controlled at (−17 ± 2) °C and (+8 ± 2) °C; and the number of cycles ranged from 50 to 300 times, respectively. The coupling effect of 150 days of chloride environment immersion and 300 freeze-thaw cycles in a chloride freeze-thaw environment was carried out, and the mechanical properties of BFRP under different environmental effects were compared and analyzed. Through SEM scanning and EDS energy spectrum analysis, the performance degradation mechanism of BFRP under different environmental effects is revealed, so as to promote the application and technology development of BFRP in complex environments.

## 2. Test

### 2.1. Specimen Design

**Figure 1:**
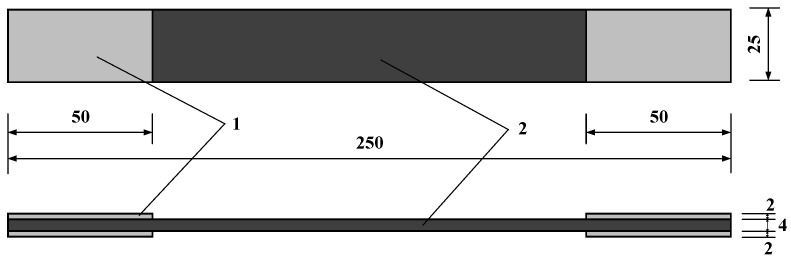
BFRP specimen (unit: mm).

In this study, the basalt fiber composite material produced by Sichuan Pawoker Mineral Fiber Products Co., Ltd. (Huaying, China) was used. The material production execution standard is GB/T31539 [18], and the relevant parameters of the specimen are shown in Table 1. A total of 120 BFRP specimens were designed, as shown in Table 2. The specimen size is shown in Figure 1.

### 2.2. Degradation Test Plan

Based on the indoor accelerated degradation test, the accelerated corrosion mechanical properties of BFRP were tested under the coupling effect of a chloride salt freeze-thaw environment. In order to compare with the single-condition environment, the coupling conditions of BFRP in the chloride freeze-thaw environment are maintained the same, that is, the concentration of NaCl solution is 5%. The concentration and PH value were examined every 7 days to ensure stability. Selecting a concentration of 5% is a conservative and harsh evaluation method, which aims to screen out materials with better performance or reveal their potential failure modes within a reasonable time. It is widely considered to be a severely accelerated corrosive environment that can simulate harsh marine environments or road environments that use a large amount of deicing salts. The soaking time was 25 days, and the specimens underwent 50 freeze-thaw cycle. Before the test, the specimens were weighed and recorded, and they were then immersed in 5% NaCl solution for 25 days. Then the specimens were taken out and put into the quick freeze-thaw box for the freeze-thaw test. The arrangement of the test specimens is shown in Table 1.

**Figure 2:**
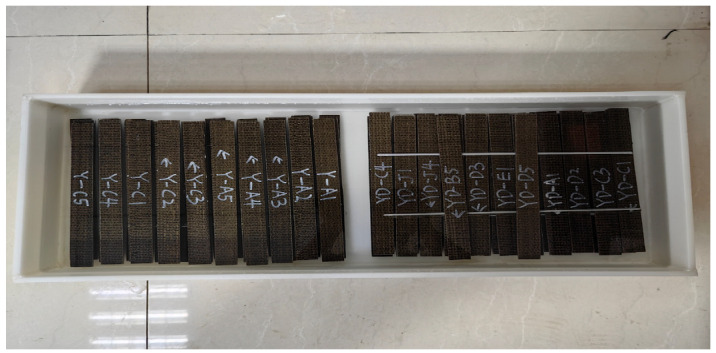
Chlorine salt corrosion schematic diagram.

The chloride corrosion test refers to ASTM C581 [19], and the test is carried out by soaking in chloride solution. The concentration of salt solution is 5% NaCl, as shown in Figure 2.

**Figure 3:**
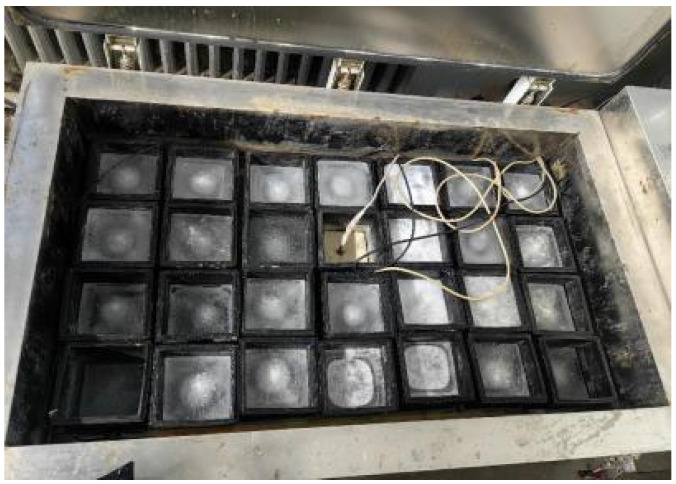
Concrete rapid freeze-thaw testing machine.

**Figure 4:**
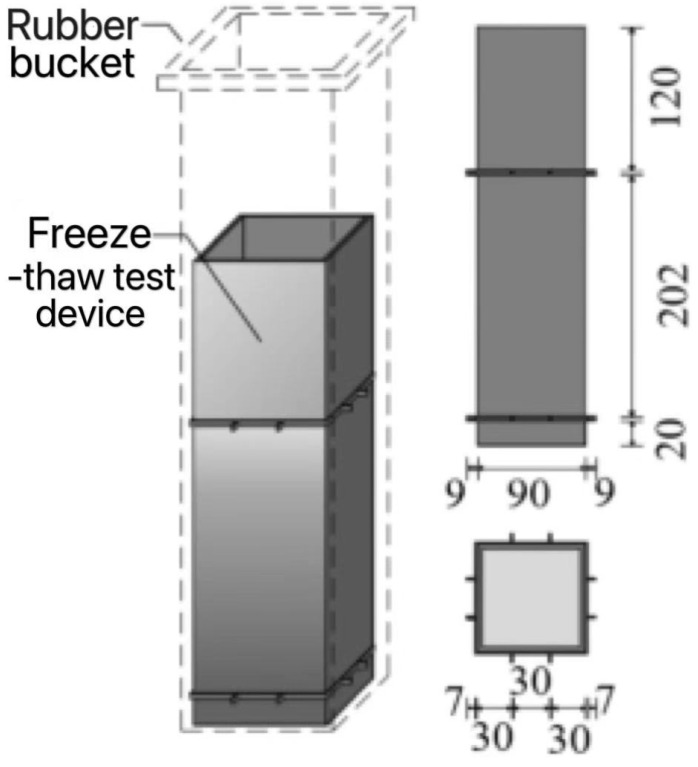
Sheet freeze-thaw cycle test device diagram.

**Figure 5:**
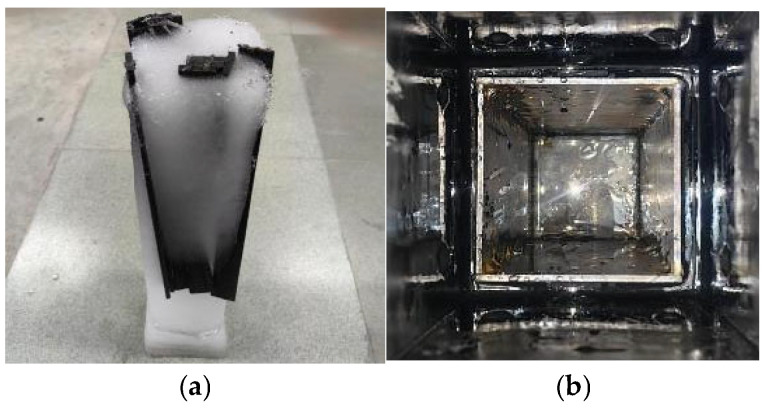
The effect diagram of freeze-thaw cycle testing after the improvement. (**a**) The water did not melt when the heating stage of BFRP was completed. (**b**) After the improvement, the water melted when the heating stage was completed.

Freeze-thaw cycles, according to the ASTM D7957’ Guideline [20] for performance test of fiber reinforced polymer matrix composites under freeze-thaw cycles, are shown in Figure 3. The freeze-thaw environment was simulated by a concrete rapid freeze-thaw test machine model KDR-V9 (Gangyuan Test Instrument Factory, Tianjin, China). The control center temperatures were (−17 ± 2) °C and (+8 ± 2) °C, respectively. The highest temperature residence time point in the freeze-thaw process is high temperature melting, and the lowest temperature residence point is low temperature freezing. The number of freeze-thaw cycles ranged from 50 to 300 times. The rubber barrel size used in the rapid freeze-thaw test machine was 110 × 110 × 500 mm. If the sheet specimen is directly put into the rubber barrel for freeze-thaw testing, it will cause ‘only freeze and not melt’ because of the large amount of water. There are problems that need to be solved urgently. In order to ensure the success and quality of the test, a device suitable for rapid freeze-thaw testing was designed, as shown in Figure 4. And its improved effect is shown in Figure 5. The specific test plan is shown in Table 1.

### 2.3. Test Plan

#### 2.3.1. Mechanical Testing Methods

**Figure 6:**
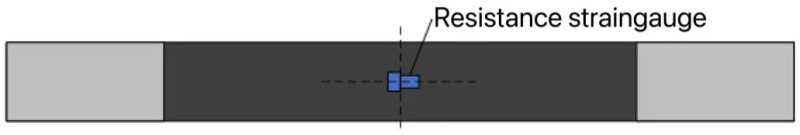
Resistance strain gauge paste position diagram.

**Figure 7:**
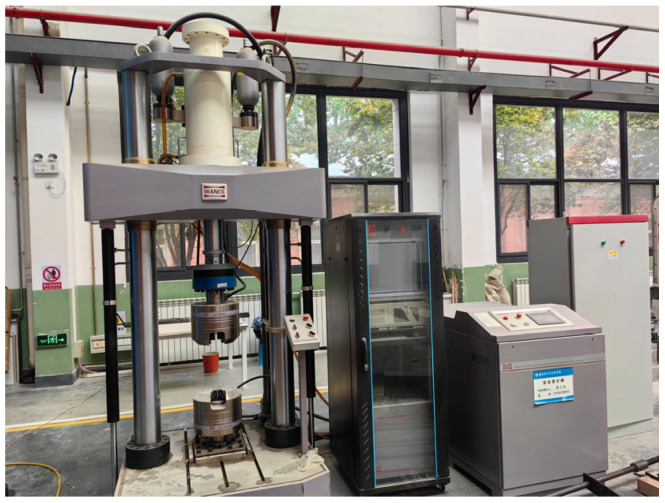
Tensile testing machine.

A tensile test according to ASTM D3039 was carried out [21]. Before the test, a resistance strain gauge was pasted horizontally and vertically in the middle of the specimen (Figure 6). In order to prevent the strain gauge from falling off or sustaining damage during the tensile process, the strain gauges were pasted on both sides. The loading method was displacement control, the rate was 2 mm/min, and continuous loading was used until the specimen was destroyed. The tensile strength, elastic modulus, elongation at break, and ultimate load were measured, and the failure modes of the specimens were recorded. The test equipment is shown in Figure 7.

#### 2.3.2. Microscopic Test Methods

1. SEM analysis

**Figure 8:**
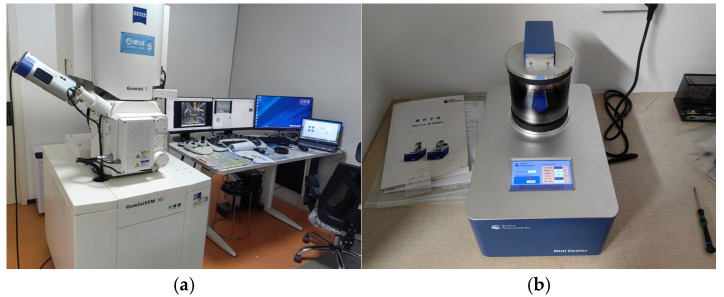
(**a**) Electron microscope scanner. (**b**) Gold spraying instrument.

In this experiment, the equipment model is Zeiss GeminiSEM 360 (Suzhou, China); the gold spraying instrument is the MiNi Coater magnetron sputtering instrument produced by Shenzhen SUP Instrument Co., Ltd. (Shenzhen, China), as shown in Figure 8. 

The BFRP specimens after corrosion were sampled as follows: the specimens with the moderate corrosion time and the longest corrosion time were selected for SEM scanning, as shown in Table 3.

2. EDS analysis

EDS spectrum analysis can be combined with SEM to obtain the distribution information of each element on the surface of the specimen in real time. EDS provides detailed data on element composition, and it further understands the law of element migration during corrosion and its influence on the properties of BFRP materials. EDS energy spectrum analysis specimens were analyzed in four groups, as shown in Table 4.

## 3. Test Phenomena and Analysis

### 3.1. Experimental Phenomena

#### 3.1.1. Corrosion Phenomenon

1. Chlorine salt environment corrosion phenomenon

**Figure 9:**
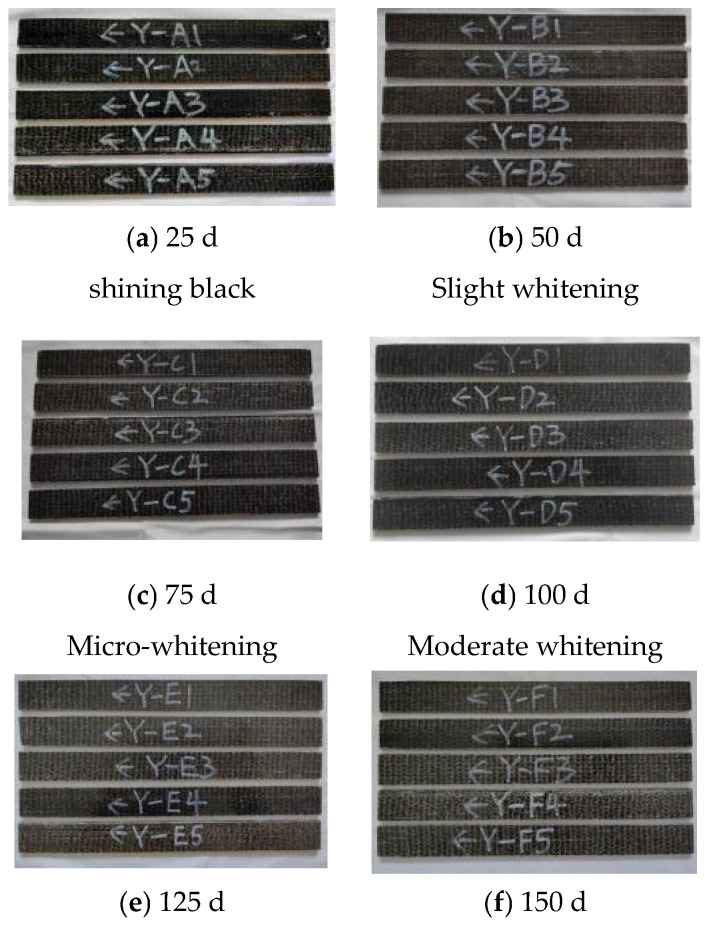
Changes in the surface properties of BFRP sheets in chloride environment.

The details of the surface corrosion of the specimen are shown in Figure 9.

It can be seen from the figure that the changes in the BFRP specimens accelerated after corrosion by chloride salt. Through the observation of different corrosion time nodes, it can be seen that with the passage of time, the surface of BFRP specimens gradually lost its luster and whitened, but the overall structure remained intact.

2. Freeze-thaw damage phenomenon

**Figure 10:**
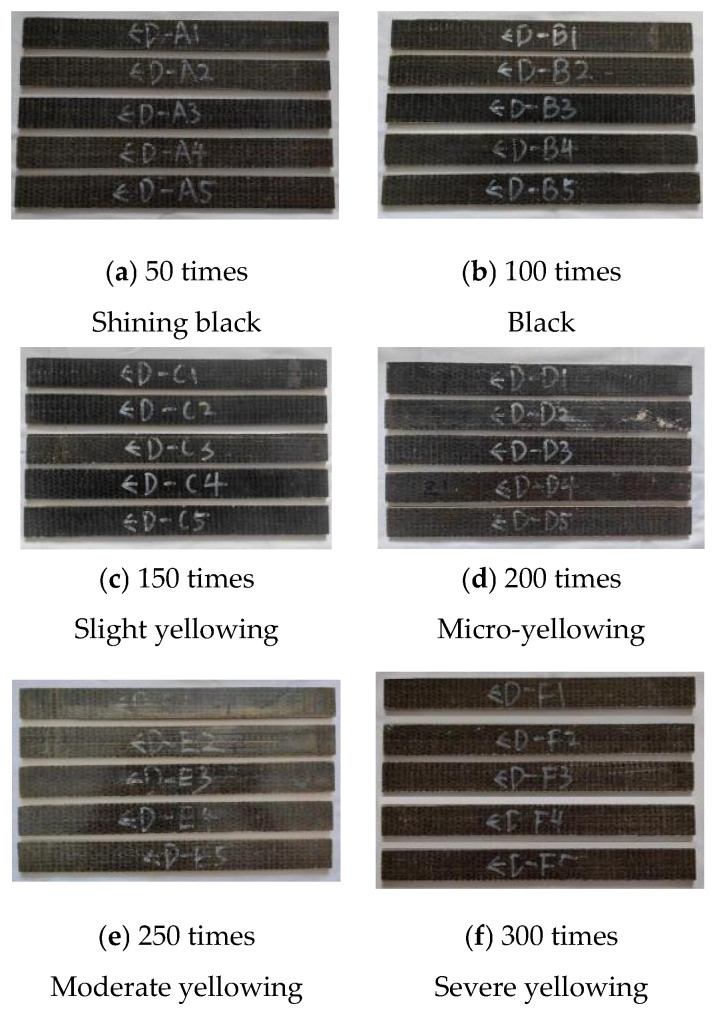
Changes in the surface properties of BFRP sheets under freeze-thaw cycles.

The details of the surface corrosion of the specimen are shown in Figure 10.

After 300 freeze-thaw cycles, the surface of the BFRP specimen D-A → D-F changed significantly. At the initial stage of freezing–thawing, the surface changes of D-A and D-B were not obvious, and there were no obvious depressions, blisters, cracks, etc., still maintaining the original bright black; midway through freezing and thawing, the D-C and D-D of the specimen gradually yellowed, and the surface of the specimen was smooth and not affected by rust; when the freeze-thaw entered the final stage, the yellowing of D-E and D-F became increasingly more serious, and there was sporadic whitening at the junction of fiber and resin. Yellowing may be the color caused by the deterioration of the resin material. However, the overall structure of the BFRP specimen was well preserved, and no serious physical damage occurred.

3. Chloride freeze-thaw environment coupling corrosion phenomenon

**Figure 11:**
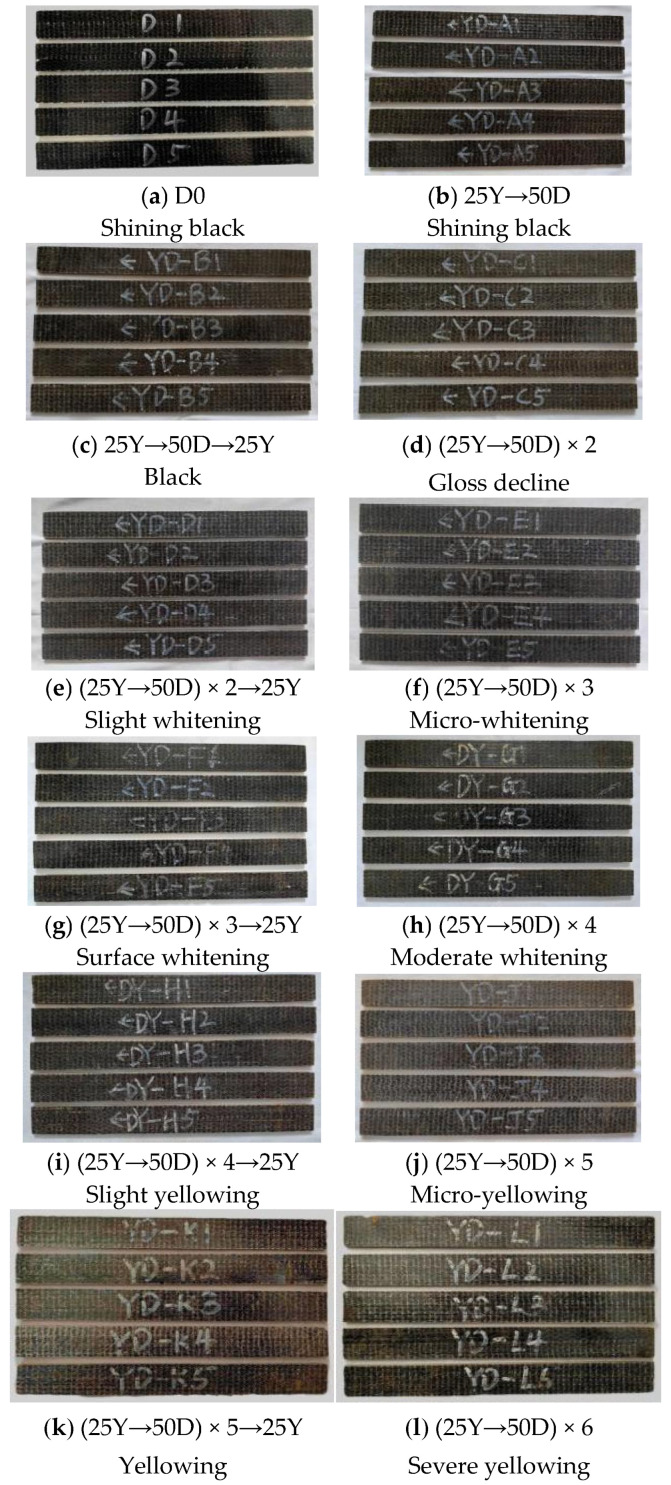
Changes in the surface properties of BFRP sheets under the coupling effect of a chloride-freeze-thaw environment.

The details of the surface corrosion of the specimen are shown in Figure 11.

Under the coupling effect of chloride salt and a freeze-thaw environment, the surface of BFRP specimens changed significantly. The specific performance is as follows: the surface of the specimens under the coupling environment was similar to that under the action of the chloride salt environment, and the glossiness decreased slightly. In YD-D specimens, the interface between the surface fiber and the resin displayed a whitening phenomenon; hallway through the coupling, the color gradually became pale yellow, and the whitening phenomenon was further aggravated. At the end of the coupling stage, the whole specimen was light yellow, and the fiber white was particularly obvious. With increasing corrosion time, the surface gloss of BFRP specimens gradually decreased, and the whitening phenomenon became increasingly more obvious, but no serious physical damage occurred.

#### 3.1.2. Loading Test Phenomenon

**Figure 12:**
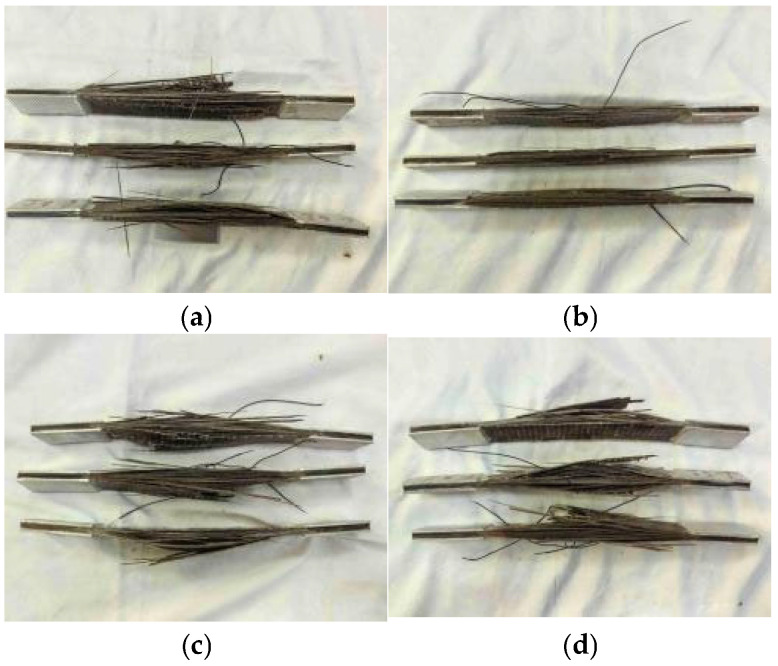
The change in tensile failure morphology of BFRP sheets. (**a**) Uncorroded control group failure mode. (**b**) The failure mode of BFRP under the action of chloride environment. (**c**) The failure mode of BFRP under freeze-thaw cycles. (**d**) Chlorine salt and freeze-thaw environment coupling.

The tensile failure pattern of the sheets is shown in Figure 12.

At the initial stage of loading (about 30%~40% of the ultimate bearing capacity), there is no obvious deformation on the surface of the specimen, and the displacement display value of the loading device increases linearly and slowly, indicating that the basalt fiber composite sheet is in the elastic stage at this time. Halfway through the loading test (about 60%~70% of the ultimate bearing capacity), as the displacement continues to increase, the surface of the specimen changes; the fiber interweaving point gradually shows a whitening phenomenon. It is preliminarily judged that the interface between the fiber and the matrix may begin to appear under the load. There is a slight separation trend, the local stress concentration of the matrix resin leads to the change in its internal microstructure, the specimen as a whole has not lost its bearing capacity, and the material is in the elastic-plastic transition stage; in the later stage of loading (about 90%~95% of the ultimate bearing capacity), when the load is close to the ultimate bearing capacity of the specimen, the test phenomenon becomes more and more obvious. The whitening area on the surface of the sheet expands rapidly and connects with each other to form an obvious damage area. Some fibers begin to break and emit a crisp ‘spray’ fracture sound. The tensile failure mode is explosive failure, and the fibers at the fracture are roughened and divergent. At the fracture, it can be clearly seen that the basalt fiber is pulled out from the matrix, and the resin coating on the fiber surface shows an obvious shedding phenomenon. Finally, the sheet loses its bearing capacity, and the test ends.

Under the effect of a chloride environment, freeze-thaw cycle, and a combination of the two, the appearance of BFRP is slightly different after the tensile test, as shown in Table 5.

### 3.2. Comparison of Different Mechanical Indexes

#### 3.2.1. Mechanical Properties of BFRP Sheets in Different Environments

**Figure 13:**
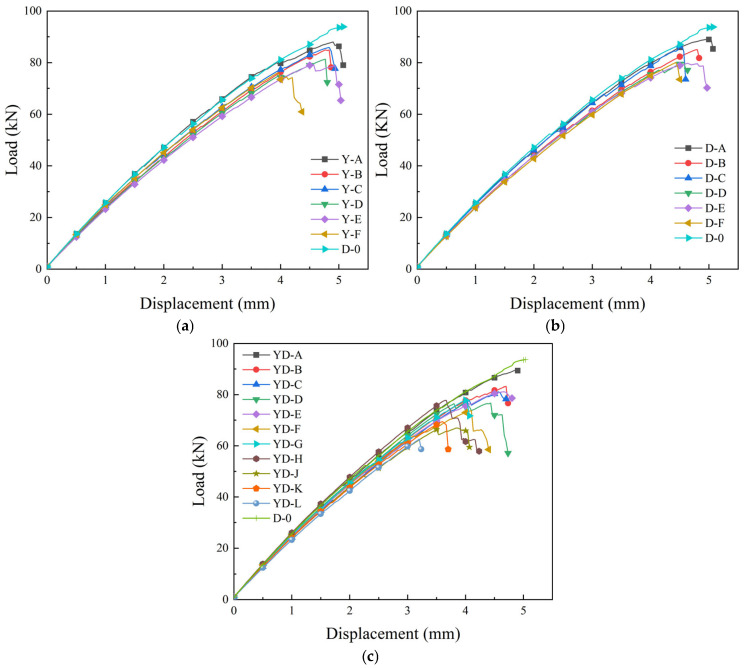
Load–displacement curves of BFRP specimens under different environmental conditions. (**a**) Chloride environment; (**b**) freezing and thawing cycle; (**c**) coupling effect of the chloride freeze-thaw environment.

The load–displacement curves of BFRP specimens under different environmental conditions are shown in Figure 13.

It can be seen from the load–displacement curve of Figure 13a from 0 to 150 days of corrosion that although there are some differences in the size of each specimen, with the increase in corrosion time, the five specimens in each stage generally show a downward trend, from the maximum load of 0 days about 80~85 kN, down to 150 days about 70~80 kN (down about 12%).

It can be seen from the load–displacement curve of 0–300 freeze-thaw cycles in Figure 13b that it displays typical brittle material characteristics, and there is no obvious plastic deformation stage.

With the increase in the number of freeze-thaw cycles, the peak load showed a downward trend. From the maximum load of about 80~90 kN in the early stage of freeze-thaw to 70~80 kN after 300 freeze-thaw cycles (a decrease of about 16%), it shows that the tensile strength of BFRP is gradually degraded.

It can be seen from the load–displacement curve of Figure 13c after the coupling effect of the chloride freeze-thaw environment that it still shows characteristics of brittle materials. With the increase in the coupling time, the peak value of the load–displacement curve shows a downward trend. In the initial stage of the test, the maximum load is about 80~90 kN; the late value decreased to 60~70 kN (decreased by about 23%). It shows that the tensile strength of BFRP continues to decay, and the decrease is significantly higher than that of a single environmental corrosion.

#### 3.2.2. Comparative Analysis of Tensile Strength

**Figure 14:**
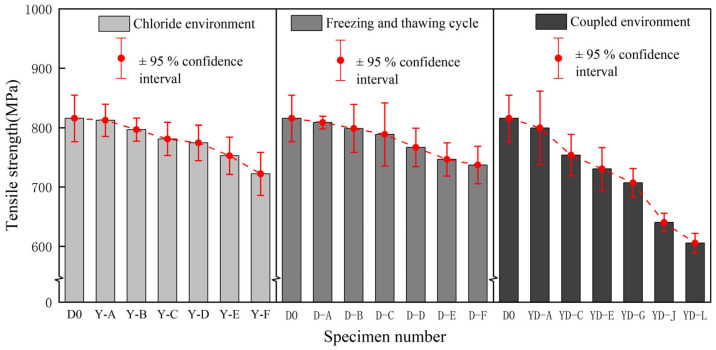
Comparison of tensile strengths of BFRP sheets under different environments.

In Figure 14, the tensile strength degradation trend of BFRP sheets under different environments is shown. Table 6 shows the specific value of the tensile strength of BFRP sheets under different environments.

The following can be seen from the chart: (1) The tensile strength of BFRP sheets decreased significantly with the increase in chloride corrosion time. The tensile strength decreased slowly in the initial stage of corrosion (25~75 days). With the increase in corrosion time (100~150 days), the tensile strength decreased rapidly, but after 150 days of chloride corrosion, the tensile strength decreased by 11.46%, with an overall decrease of less than 15%, indicating that BFRP has good resistance to chloride corrosion. (2) The tensile strength of BFRP sheets decreased gradually with the increase in freeze-thaw cycles. In the early stage of the freeze-thaw cycle, the tensile strength decreases more gently; in the later stage, the decline increased significantly, and the final decline was 9.62%. Compared with the chloride environment, the performance degradation of BFRP in freeze-thaw cycles is relatively light, indicating that it still has good durability under freeze-thaw cycles. (3) The tensile strength of BFRP specimens under the coupling effect of a chloride freeze-thaw environment showed a continuous downward trend. Compared with chloride salt and freeze-thaw cycle, the decrease rate and decrease range of the coupling effect are significantly accelerated. When YD-L is coupled, the tensile strength of the specimen decreases by 25.73%. Compared with 11.46% in the chloride salt environment and 9.62% in the freeze-thaw cycle, the decrease range increases by nearly two-fold, showing an obvious ‘1 + 1 > 2’ effect.

#### 3.2.3. Comparative Analysis of Elastic Modulus

The results of this study show that the tensile strength loss of BFRP is as high as 25.7% under the coupling effect of chloride salt freeze-thaw. This degradation level serves as an important warning for the actual engineering lifespan. First, in structural design, FRP materials usually enjoy a certain safety factor (for example, 0.5–0.6 of the ultimate strength of the material is taken as the allowable stress). The strength degradation observed in this study has significantly eroded this safety margin and may affect the long-term safety of the structure. Secondly, the laboratory-accelerated test conditions are equivalent to years of aging in the actual environment. The order of performance degradation degree (coupling > freeze-thaw > chloride salt) shows that the effective service life of BFRP members may be much shorter than that in mild environments in coupled environments such as bridges or coastal areas where deicing salts are used in cold regions.

**Figure 15:**
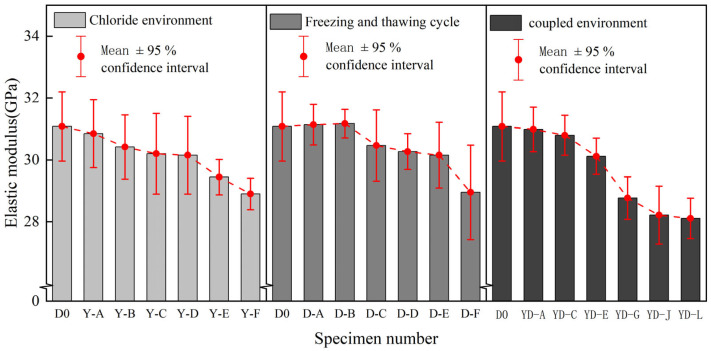
Comparison of elastic modulus of BFRP sheets under different environments.

Figure 15 shows the decreasing trend of the elastic modulus of BFRP sheets under different environmental effects. Table 7 shows the specific value of the elastic modulus of BFRP in different environments.

The following can be seen from the chart: (1) The elastic modulus of BFRP sheets under the action of a chloride salt environment showed a downward trend as a whole, the decrease was more obvious in the later stage of corrosion (125 days~150 days), and the dispersion showed a convergence trend in the later stage. The elastic modulus decreased slightly in the early stage of chloride corrosion. After 150 days of corrosion, the elastic modulus decreased by 7.02%. (2) In the figure, the elastic modulus increases slightly when the number of freeze-thaw cycles is small, but the elastic modulus begins to decrease after multiple freeze-thaw cycles. The reason is that the resin base in the material gradually becomes brittle due to the sudden decrease in temperature in the early stage of freeze-thaw so that the stiffness of the specimen increases. With the increase in the number of freeze-thaw cycles, the BFRP specimen shows a damage accumulation effect so that the elastic modulus increases first and then decreases. The elastic modulus of BFRP decreased most seriously (9.62%) during the 300th freeze-thaw cycle, but the range was within 10%. (3) At the initial stage of coupling, the elastic modulus decreased slowly, while the elastic modulus of the specimen YD-G decreased in a ‘cliff-like‘ manner, and the tensile strength also decreased in a ‘cliff-like‘ manner. This phenomenon may be due to the sudden drop in the temperature of the freeze-thaw cycle, which increases the stiffness of the resin and is coupled with the chloride environment. However, with the deepening of corrosion, the internal damage of the material gradually accumulates, resulting in a sudden decrease in the elastic modulus. In the YD-J stage of the specimen, the decrease in elastic modulus tends to be stable, the decrease rate is maintained within 0.2%, and the material stiffness remains relatively stable. At the end of the coupling test, the elastic modulus of BFRP specimens decreased by 9.55%, which was slightly higher than that under a chloride environment (7.02%) and freeze-thaw cycle (6.85%).

#### 3.2.4. Comparative Analysis of Elongation at Break

**Figure 16:**
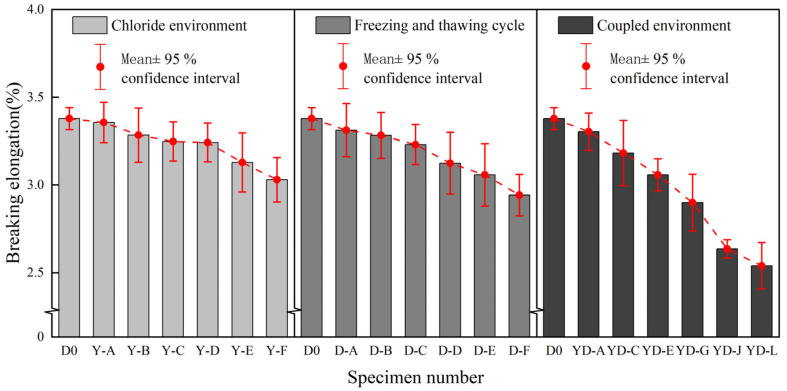
Comparison of elongation at break of BFRP sheets under different environments.

In Figure 16, the decreasing trend of elongation at break of BFRP sheets under different environments is shown. Table 8 shows the specific value of elongation at break of BFRP under different environments.

The following can be seen from the chart: (1) In the early stage of chloride action (0~100 days), the decline is not obvious, which is similar to the decline trend of elastic modulus, and the decline is more intense in the later stage, indicating that the ductility and toughness of the material are reduced after long-term corrosion. The elongation at break of the specimen decreased by 10.27% after 150 days in the chloride environment. (2) With the increase in the number of freeze-thaw cycles, the elongation at break of the specimen continues to decrease. In the early stage of the freeze-thaw cycle, the overall decline is relatively gentle, and the decline rate of each group of specimens is basically maintained at about 2%. The number of freeze-thaw cycles increased to 250 and 300, and the rate of decline at this stage increased to about 3%. After 300 freeze-thaw cycles, the elongation at break decreased by 12.95%. (3) After 150 days in a chloride environment and 300 freeze-thaw cycles, the elongation at break showed a downward trend as a whole, and the initial decline was relatively slow, and the later period was more serious. At the initial stage of the coupling effect (before the specimen YD-E), the elongation at break decreased by about 3%, but the elongation at break decreased by more than 5% at the later stage of the coupling effect (after YD-G), until 150 days. The elongation at break decreased by 24.81% after the coupling of a chloride environment and 300 freeze-thaw cycles. Compared with the change in elongation at break in a single-condition environment, the elongation at break of BFRP decreases significantly under the coupling effect of the two, showing more serious brittleness characteristics.

The degradation experiment period of this study was 150 days of chlorine salt corrosion, 300 freeze-thaw cycles, and their coupling effect. During this period, the performance degradation of the material did not reach the platform period (Figure 12, Figure 13 and Figure 14). Based on its tensile strength, elastic modulus, elongation at break, and hydrolysis mechanism and by observing its downward trend, it can be reasonably speculated that the degradation process of the material will continue for a longer exposure time until complete degradation or loss of function. However, this inference needs to be verified by longer-term experiments.

## 4. Analysis of the Microstructure and Degradation Mechanism

### 4.1. Micro-Morphology Test Analysis

**Figure 17:**
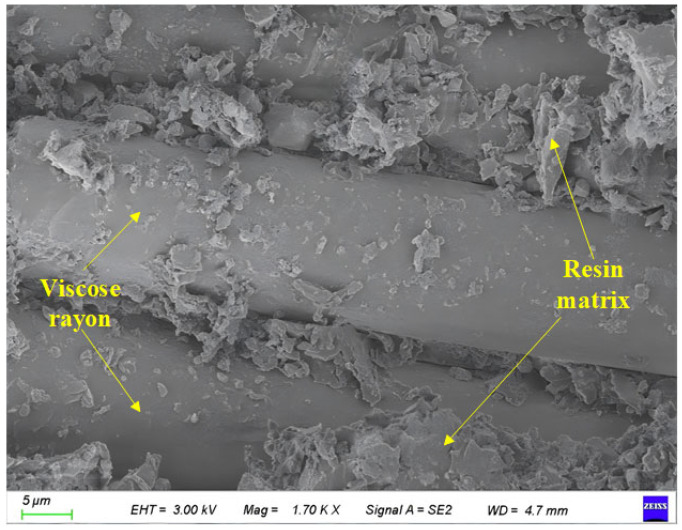
Local images of the control group.

Figure 17 shows the SEM scanning results of the surface of the BFRP specimen in the control group. Its magnification is 1700 times.

It can be seen from Figure 17 that the basalt fiber is cylindrical and closely combined with the epoxy resin-based material. The two show viscosity as a whole and no dispersion occurs. At the same time, the surface of the fiber is smooth, only a small amount of epoxy resin is attached, and there are no defects such as holes and depressions on the surface.

**Figure 18:**
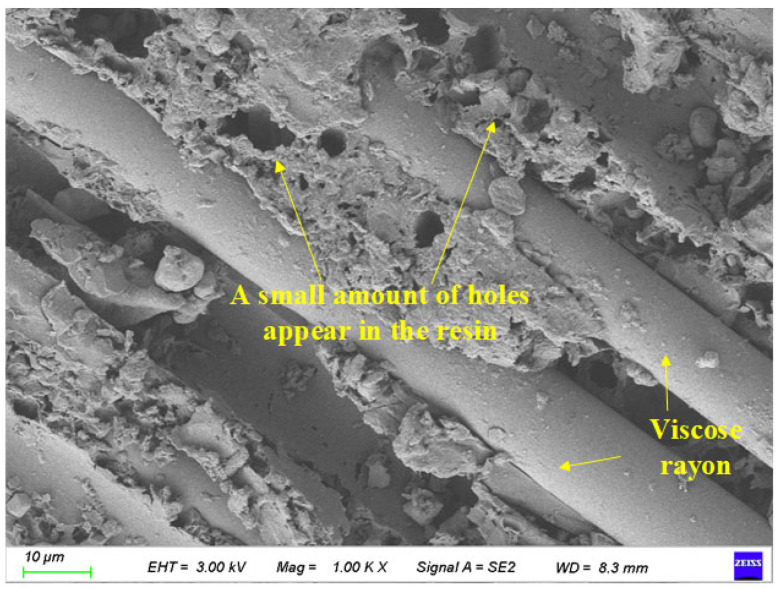
Local image of the specimen after 75 days of chloride action.

Figure 18 shows the surface characteristic scanning results of BFRP specimens after 75 days of chloride action. Its magnification is 1000 times.

It can be seen from Figure 18 that after 75 days in the chlorine salt environment, the resin base attached to the surface of the fiber was significantly reduced compared with the control group. A large number of holes appeared in the epoxy resin matrix and showed a fragmented state. Most of the craters are distributed in the epoxy resin-based region between each fiber, and the distribution is relatively uniform.

**Figure 19:**
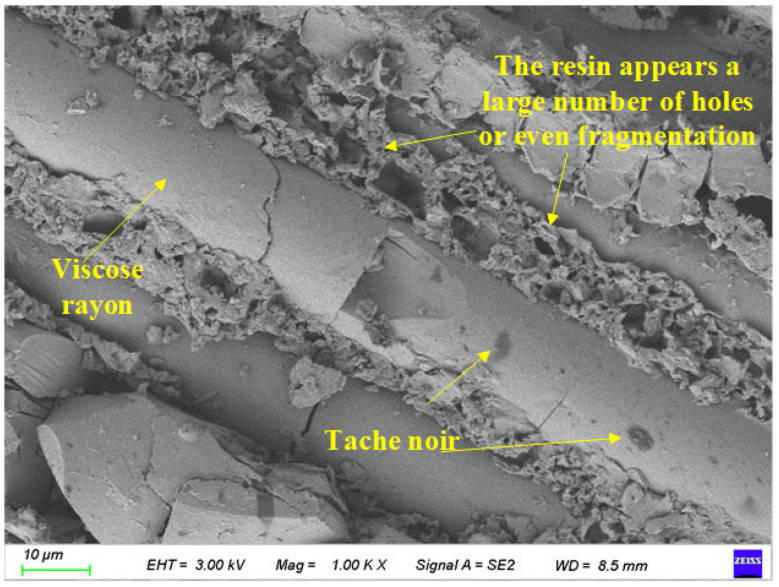
Local image of the specimen after 150 days of chloride action.

Figure 19 shows the surface characteristic scanning results of BFRP specimens after 150 days of chloride action. Its magnification is 1000 times.

It can be seen from Figure 19 that compared with the soaking time of 75 days, the degree of fragmentation of the resin matrix between the fiber filaments was significantly increased, and the ‘sponge-like‘ characteristics were gradually presented. The voids in the epoxy resin matrix between the filaments were further increased, and the surface gradually changed from the previous relatively smooth state to the ‘sponge‘ shape. Compared with the case after 75 days of corrosion, many black spots appeared on the surface of the fiber. It is speculated that the reason may be that the chloride crystal in the solution precipitates and adheres to the surface of the fiber.

**Figure 20:**
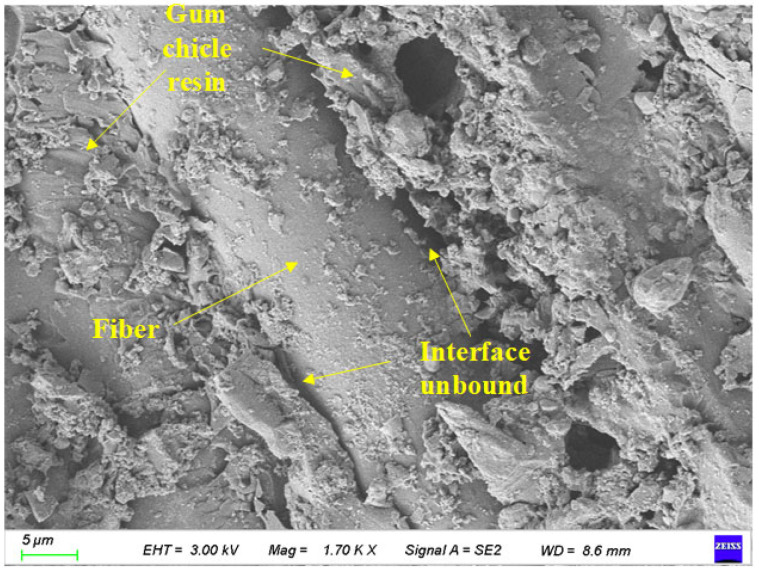
Local images of 150 freeze-thaw cycles of specimens.

Figure 20 shows the scanning results of the surface characteristics of BFRP specimens after 150 freeze-thaw cycles. Its magnification is 1700 times.

According to Figure 20, compared with the control group, it can be found that the surface of the fiber and the resin matrix show no obvious change in essence, and there is still a small amount of resin matrix material attached to the surface of the fiber. There is a gap between some fiber filaments and the resin matrix, and the number of fiber filaments exposed on the surface is relatively reduced. There are still some strip grooves on the resin surface, and the fiber filaments have a tendency to fall off from the resin matrix material.

**Figure 21:**
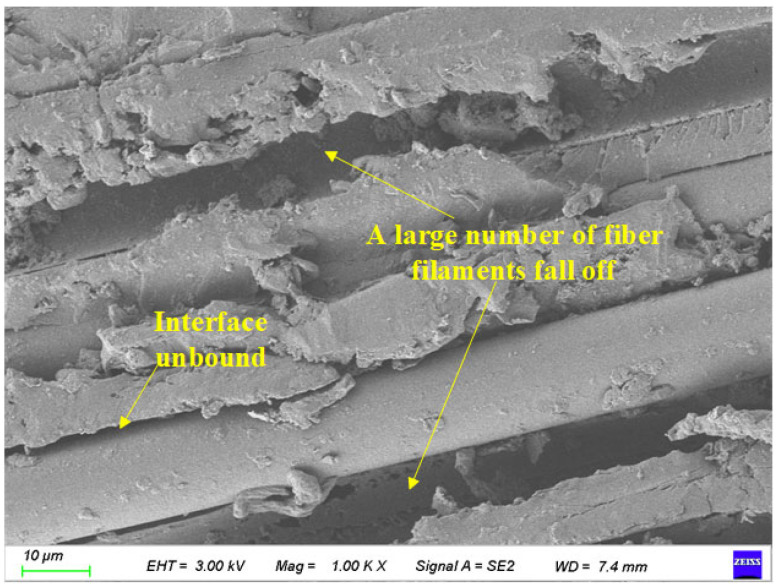
Local images of 300 freeze-thaw cycles of specimens.

As shown in Figure 21, the surface characteristics of BFRP specimens were scanned using an electron microscope after 300 freeze-thaw cycles. Its magnification is 1000 times.

It can be seen from Figure 21 that compared with 150 freeze-thaw cycles, when BFRP undergoes 300 freeze-thaw cycles, the shedding of fiber filaments is more serious, a large number of fiber filaments are detached from the resin, and more strip grooves appear, but a small amount of resin is still attached to the unshed fiber filaments.

**Figure 22:**
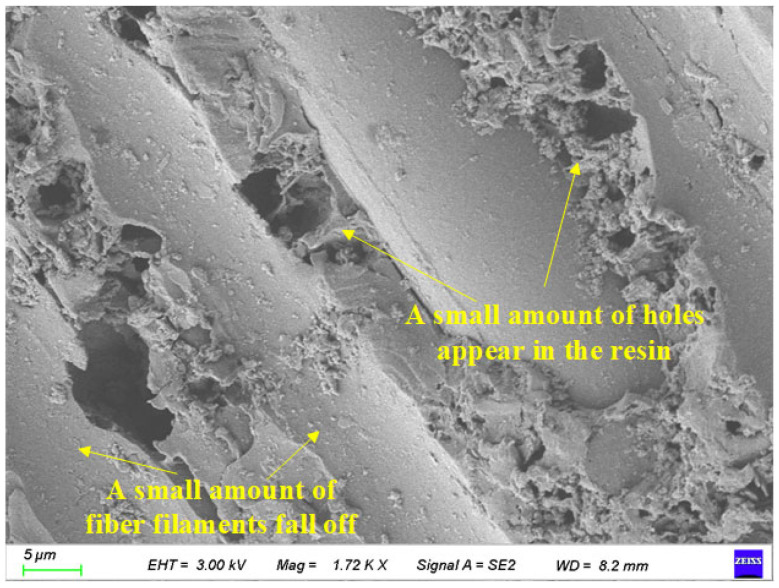
Local images of specimens under the coupling effect of 75 days of chloride corrosion and 150 freeze-thaw cycles.

As shown in Figure 22, the surface characteristics of BFRP specimens after 75 days in a chloride environment and 150 freeze-thaw cycles were scanned by electron microscopy. Its magnification is 1700 times.

It can be seen from Figure 22 that under the action of chloride environment, the number of resin bases attached to the surface of the fiber decreases, and holes appear in the resin between the fibers. With the synergistic effect of freeze-thaw cycles, the gap between the fiber and the resin matrix is further increased so that the chloride solution can penetrate into the specimen more easily, which aggravates the corrosion depth and eventually leads to the continuous decrease in material strength. At the same time, a small amount of fiber filaments fell off on the surface of the specimen.

**Figure 23:**
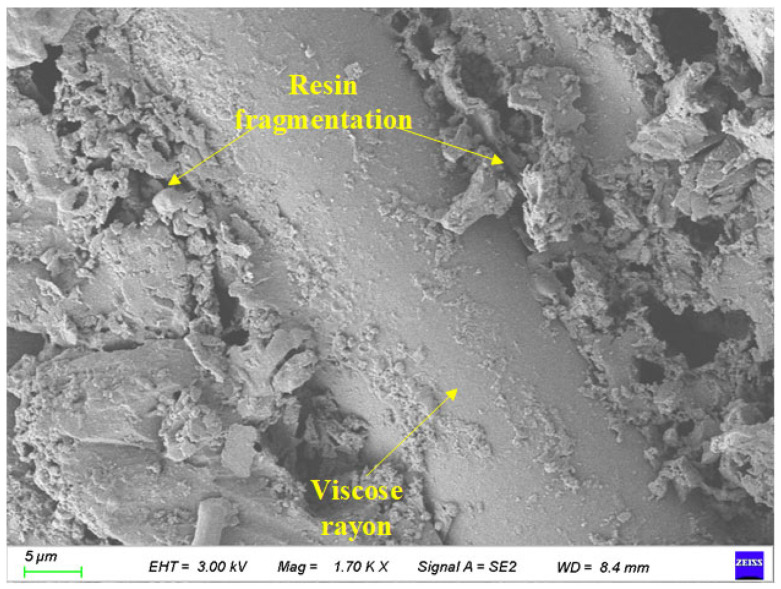
Local images of specimens under the coupling effect of 150 days of chloride corrosion and 300 freeze-thaw cycles.

As shown in Figure 23, the surface characteristics of BFRP specimens under the coupling action of 150 days in a chloride environment and 300 freeze-thaw cycles are scanned by electron microscopy. Its magnification is 1700 times.

It can be seen from Figure 23 that a large number of fiber filaments are separated from the resin, leaving many grooves formed by the shedding of fiber filaments on the surface of the material. At the same time, the gap between the fiber filaments and the resin matrix is further expanded. With the increase in the action time of the chloride environment, the number of pores in the resin matrix material between the fiber filaments increases significantly, showing obvious ‘sponge-like‘ characteristics. Black spots also appeared on the surface of the fiber.

In summary, compared with the deterioration characteristics of BFRP in a single-condition corrosion environment, its deterioration under the coupling of a chloride freeze-thaw environment is mainly reflected in the following three aspects:

(1) Superposition of corrosion characteristics: When BFRP is in the coupling of the chloride freeze-thaw environment, it shows the characteristics of a chloride environment and freeze-thaw cycle at the same time. Referring to the test results in a single-condition environment, it can be inferred that the chloride environment reduces the number of resin bases on the surface of the fiber, and the resin base shows holes and is fragmented. The freeze-thaw cycle increases the gap between the fiber and the resin matrix, and the phenomenon of fiber shedding is aggravated. Under the coupling effect of a chloride freeze-thaw environment, the internal structure of the specimen damage is more complex and serious, which leads to a faster decrease in material strength.

(2) The synergistic deterioration of the corrosion process: Under the action of a chloride environment, the holes and fragmentation of the resin matrix of BFRP make it easier for the chloride solution to penetrate into the specimen. The increase in the gap between the fiber and the resin matrix caused by the freeze-thaw cycle provides more channels for the penetration of the chloride solution. These two corrosion processes promote each other, accelerate the deterioration process of the internal structure of the material, and then accelerate the reduction of the durability of the material. With the increase in coupling time, this synergistic deterioration effect becomes increasingly more significant, and the phenomenon of fiber falling off and resin base breaking become increasingly more serious.

(3) Microstructure change mechanism: The chloride environment reacts with some components in basalt fiber, and it then destroys the fiber structure; the difference in the thermal expansion and contraction caused by freeze-thaw cycles weakens the bonding force between the fiber and the resin matrix. In the coupling environment, the chemical-physical coupling mechanism is intertwined, which changes the microstructure of the material, resulting in the fiber and resin matrix losing its synergistic ability, and the integrity of the material is seriously damaged. This series of changes will eventually have a negative impact on the practical application performance and service life of materials in engineering.

### 4.2. EDS Analysis

**Figure 24:**
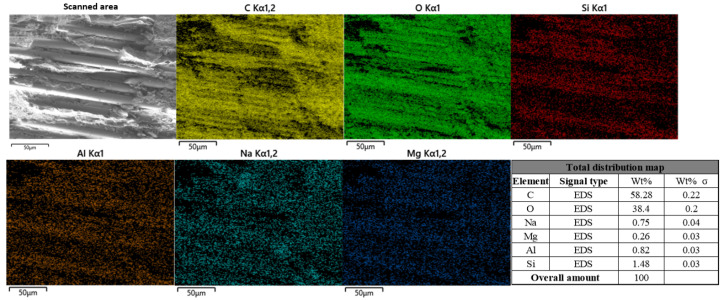
Energy spectrum analysis results of BFRP sheet uncorroded control group specimens.

Figure 24 shows a control group specimen analyzed by EDS.

It can be seen from the figure that the main elements in the material can be detected by EDS analysis as C, O, Na, Mg, Al, and Si. It can be seen from the analysis results that the distribution of C and O elements on the surface of the material is relatively uniform, accounting for 58.25% and 38.4%, respectively, and the content is high. These elements mainly come from the epoxy resin matrix. A small amount of Na was also detected in the picture, but because the control group was not exposed to the chloride environment, these Na traces may be the impurities introduced during the production process. Si, Al, and Mg are mainly distributed in the fibers.

**Figure 25:**
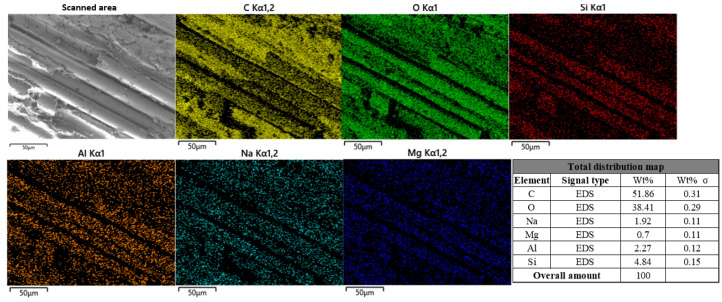
Specimen energy spectrum analysis results of BFRP sheets under chloride salt action.

In Figure 25, the results of the low-scale overall surface scanning of the specimen surface under the action of a chloride environment by EDS analysis are shown.

It can be seen from the figure that the proportion of C is about 51.86%. Compared with the uncorroded control group, the distribution density of C on the fiber is significantly reduced. The proportion of O is about 38.41%, which is similar to that of the uncorroded control group. The distribution of Na on the fibers in the map is more obvious and its proportion is about 1.92%, which is significantly higher than that of the control group (0.75%). The distribution of Si, Al, and Mg on the fiber wire increased compared with the uncorroded control group, and the proportions of these elements were 4.84%, 2.27%, and 0.7%, respectively.

**Figure 26:**
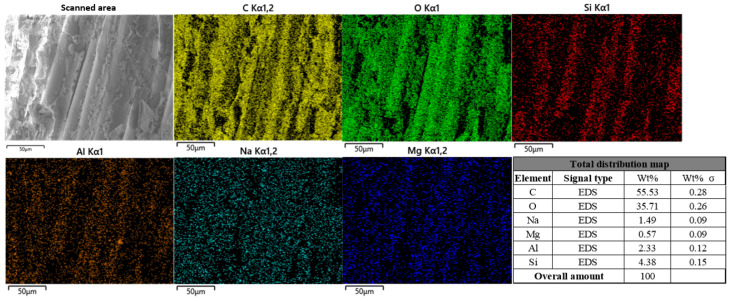
Energy spectrum analysis results of BFRP specimens under freeze-thaw cycles.

Figure 26 shows a low-power overall surface scan of the surface of the freeze-thaw cycle specimen using EDS analysis.

From the diagram, it can be seen that the proportion of C is about 55.53%, which is lower than that of the control group, and the distribution density on the fiber is reduced. The proportion of O is about 35.71%, and the concentration of O in the resin area is also reduced, indicating that the freeze-thaw cycle has an impact on the resin matrix. The distribution of Na in the figure is more obvious, accounting for about 1.49%, which is significantly higher than that of the uncorroded control group. Mg accounts for about 0.57%, Al accounts for about 2.33%, and Si accounts for about 4.38%. The increase in these elements indicates that the metal oxides in the fibers are dissolved.

**Figure 27:**
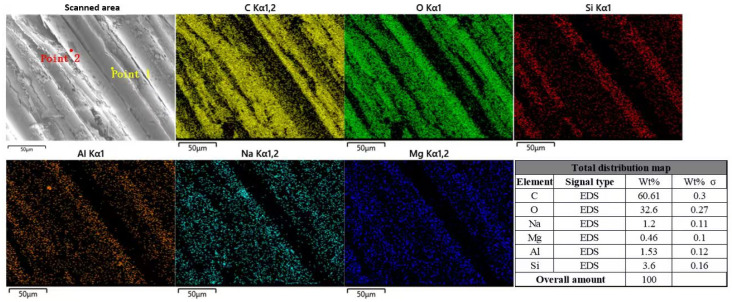
The energy spectrum analysis results of BFRP under the coupling effect of chloride salt and freeze-thaw environment.

As shown in Figure 27, EDS was used to analyze the BFRP specimens under the coupling action of a chloride freeze-thaw environment.

It can be seen from the figure that the proportion of C is about 60.61%. In the fiber area, the distribution of C is obviously sparse, while C distribution in the resin area is still dense. The proportion of O is about 32.6%, which is lower than that of the uncorroded control group. The distribution of O in the resin area is relatively sparse. The proportion of Na is about 1.2%. At point 2 in the figure, the concentration of Na is extremely dense in the ‘black spot‘ area on the surface of the fiber, which is the corrosion pit on the surface of the fiber. The distribution of Si, Al, and Mg on the fibers changed compared with the uncorroded control group, and the proportions of these three elements were 3.6%, 1.53%, and 0.46%, respectively.

### 4.3. Deterioration Mechanism Analysis

#### 4.3.1. Degradation Mechanism of the Resin Matrix

**Figure 28:**
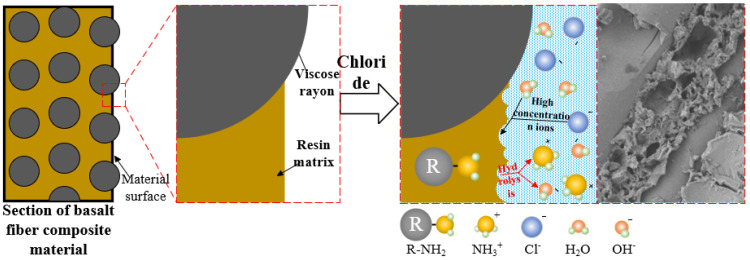
Degradation mechanism of BFRP resin.

As shown in Figure 28, the chloride solution penetrates into the interior of the material through microcracks or pores in the resin matrix and hydrolyzes with the amine curing agent in the epoxy resin, destroying the cross-linked network structure of the resin and resulting in a decrease in the mechanical properties of the resin matrix.

The ethylene diamine aliphatic amine curing agent (molecular formula is R-NH2, R is an organic group) with the highest content in the oxygen resin material is analyzed as follows:

(1) In sodium chloride solution, water molecules are abundant, and amine curing agent (R-NH_2_) undergoes proton transfer reaction with the water molecules. The hydrogen–oxygen bond in the water molecule has a certain polarity, and the hydrogen atom has a partial positive charge. At this time, the nitrogen atom of the amine combines with the hydrogen proton in the water molecule to form an ammonium ion (R-NH_3_^+^). At the same time, the water molecule loses the proton to form a hydroxide ion (OH^−^). The hydrolysis reaction equation is as follows:

$${R-NH}_{2}+H_{2}O⇌{R-NH}_{3}^{+}+{OH}^{-}$$

In essence, this reaction embodies the acid–base proton theory, in which amines play the role of bases and accept the protons provided by the water molecules.

(2) Sodium chloride (NaCl) is completely ionized in the solution to generate sodium ions (Na^+^) and chloride ions (Cl^−^). The chloride ion in the chloride salt solution has strong nucleophilicity, and the molecular structure of the epoxy resin contains parts that are vulnerable to nucleophilic attacks such as ether bonds, thereby promoting the hydrolysis reaction of the epoxy resin. The chloride ion also destroys weak interactions such as hydrogen bonds in the epoxy resin curing network, making the structure of the cured product relatively loose, which is more conducive to the contact between water molecules and epoxy resin molecules and accelerates the hydrolysis process.

(3) The ammonium ions (R-NH_3_^+^) and hydroxide ions (OH^−^) produced by the hydrolysis of amine curing agents change the chemical environment of the system. Among them, the hydroxyl ion (OH^−^), as a strong catalyst, further accelerates the hydrolysis reaction of the epoxy resin, resulting in the ring opening of the epoxy group and the formation of hydroxyl (-OH).

#### 4.3.2. Interface Failure Mechanism

**Figure 29:**
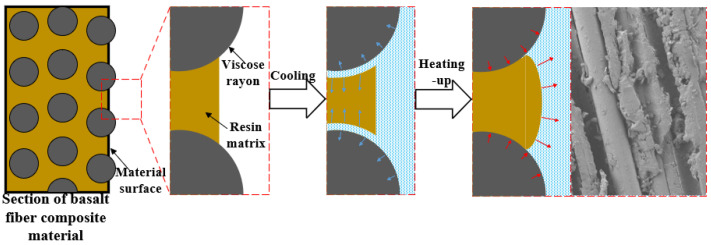
Degradation mechanism of BFRP interface failure.

There is a significant difference in the thermal expansion coefficient between the fiber filaments in BFRP and the resin matrix. In a single freeze-thaw cycle, due to the effect of thermal expansion and cold contraction, the physical deformation degree of the two is inconsistent, resulting in the expansion of the gap between the fiber and the resin matrix. This gap expansion phenomenon weakens the bonding force between the fiber and the resin matrix, seriously affects the integrity of the material, and ultimately leads to a decrease in the strength of the material, as shown in Figure 29.

Every time a freeze-thaw cycle is completed, a certain gap width will be generated between the fiber and the resin matrix, and with the increase in the number of freeze-thaw cycles, the gap will gradually increase. When it interacts with the chlorine salt solution at the same time, the amine curing agent in the resin will gradually undergo a hydrolysis reaction, resulting in holes inside the resin, and the appearance of holes further expands the gap, thereby reducing the strength of the material itself.

#### 4.3.3. Damage Mechanism of the Fiber Itself

When the basalt fiber is in a high-concentration ion solution environment, some of the metal oxides (such as magnesium oxide) may undergo ion exchange with the ions in the solution, thereby destroying the structure of the fiber. The environmental concentration of chloride salt used in this paper is 5%, which belongs to the category of higher concentration. The main corrosion mechanism in this environment is that the hydroxyl ion (OH^−^) produced by the hydrolysis of the resin-based material reacts with the silica in the basalt fiber wire. The reaction equation is as follows: ${SiO}_{2}+2{OH}^{-}\to {SiO}_{3}^{2-}+H_{2}O$.

**Figure 30:**
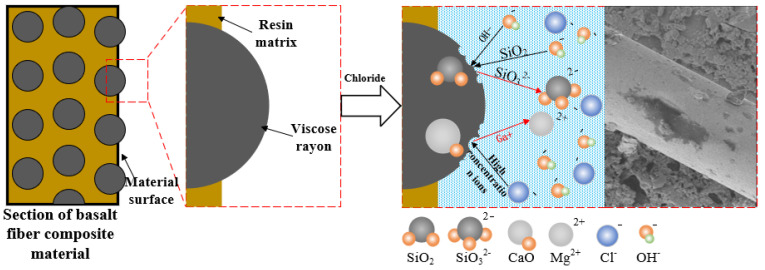
Deterioration mechanism of BFRP filaments.

After the configuration of 5% sodium chloride solution was completed, the PH value was determined. At this time, the PH value was between 7.1 and 7.2, which was neutral; after soaking BFRP in the solution for 150 days, the pH value of the solution is between 8.2 and 8.4, which is weakly alkaline. It shows that under the influence of chloride environment, the hydrolysis reaction rate of epoxy resin will be accelerated, resulting in more hydroxide ions (OH^−^), which will further aggravate the corrosion of basalt fiber filaments, as shown in Figure 30.

When BFRP experiences the coupling effect of the chloride freeze-thaw environment, it will show the characteristics of a chloride environment and freeze-thaw cycle at the same time; the chloride environment causes the hydrolysis of the resin matrix and produces holes, while the freeze-thaw cycle causes the expansion of the gap between the fiber and resin matrix, which provides more channels for the penetration of chloride solution. The chloride environment causes chemical corrosion in the fiber and resin matrix. Under the action of freeze-thaw cycles, the interface failure is caused by the difference in thermal expansion and cold shrinkage, and the overall durability of BFRP is degraded more seriously under the action of chemical and physical coupling. EDS showed that Na was densely distributed at the ‘black spot‘, and Si, Al, and Mg on the resin were significantly reduced.

The degradation mechanism of BFRP is mainly manifested as resin matrix hydrolysis, interface failure, and fiber damage due to the coupling effect of the chloride environment, freeze-thaw cycle, and chloride freeze-thaw environment. These changes reduce the overall performance of the material and shorten its service life.

## 5. Conclusions

This paper focuses on the cold and arid irrigation areas in northwest China and reveals the durability degradation law of basalt fiber reinforced polymer (BFRP) under the coupling effect of a chloride environment, freeze-thaw cycle, and chloride freeze-thaw environment. Through our research, the following conclusions are drawn.

1. The evolution of the physical and mechanical properties of BFRP under chloride, freeze-thaw, and their coupling effects was studied. The results show the following: (1) The chloride environment (5% concentration, 150 days) leads to the whitening of the fiber lap on the surface of BFRP, the salt crystals are precipitated at the cross-section cracks, and the quality is increased. The tensile strength, elastic modulus, and elongation at break decreased by 11.46%, 7.02%, and 10.27%, respectively. (2) After freeze-thaw (300 times), the surface of BFRP is yellow, the cross-section crack expands, and the quality is basically unchanged. The mechanical properties decreased by 9.62%, 6.85%, and 12.95%, respectively. (3) Under the coupling effect of chloride salt and freeze-thaw, BFRP appeared to be whitening and yellowing at the same time, the mass increased, and the mechanical properties decreased most significantly, reaching 25.73%, 9.55%, and 24.81%, respectively, indicating that the coupling effect significantly accelerated the degradation of the material properties.

2. The microscopic degradation mechanism of BFRP under chloride salt, freeze-thaw, and their coupling was analyzed by SEM and EDS. The results showed the following: In the chloride salt environment, the resin base was seriously hydrolyzed to be ‘sponge-like’, and OH was released to make the solution weakly alkaline. ‘Black spots’ and corrosion pits appeared on the surface of the fiber, and Na was enriched. In the freeze-thaw cycle, due to the difference in the thermal expansion coefficient between the fiber and the resin, the interface gap is expanded, resulting in increased fiber shedding and reduced resin attachment elements. Under the coupling effect, the freeze-thaw expansion interface promotes chloride salt infiltration corrosion, the chloride salt hydrolysis produces holes and enhances the freeze-thaw expansion effect, and the chemical and physical synergy accelerates the durability degradation of BFRP.

## Figures and Tables

**Table 1:** BFRP plate-related information.

Serial Number	Item	Content
1	Lay-up sequence	Cross section from top to bottom in the order of ‘fabric → fiber → fabric‘
2	Fiber orientation	The whole fiber is in the length direction (longitudinal) of the composite material, and some fibers in the fabric are in the transverse direction or at a certain angle (this part accounts for a small proportion)
3	Workmanship	Draw-extruding
4	Fiber volume fraction	70%
5	Whether post curing treatment is carried out	No

**Table 2:** BFRP sheet chloride freeze-thaw environment coupling arrangement table.

Environmental Category	Specimen Number	Specimen Size	Freeze-Thaw Times	5% Chlorine Salt Soaking Time	Trial Amount
Unattacked	D0	250 × 25 × 4	—	—	5
Chloride environment	BFRP-Y-A	250 × 25 × 4	—	25 d	5
	BFRP-Y-B		—	50 d	5
	BFRP-Y-C		—	75 d	5
	BFRP-Y-D		—	100 d	5
	BFRP-Y-E		—	125 d	5
	BFRP-Y-F		—	150 d	5
Freezing and thawing cycle	BFRP-D-A	250 × 25 × 4	50	—	5
	BFRP-D-B		100	—	5
	BFRP-D-C		150	—	5
	BFRP-D-D		200	—	5
	BFRP-D-E		250	—	5
	BFRP-D-F		300	—	5
Chloride freeze-thaw coupling environment	BFRP-YD-A	250 × 25 × 4	25Y→50D		5
	BFRP-YD-B		25Y→50D→25Y		5
	BFRP-YD-C		(25Y→50D) × 2		5
	BFRP-YD-D		(25Y→50D) × 2→25Y		5
	BFRP-YD-E		(25Y→50D) × 3		5
	BFRP-YD-F		(25Y→50D) × 3→25Y		5
	BFRP-YD-G		(25Y→50D) × 4		5
	BFRP-YD-H		(25Y→50D) × 4→25Y		5
	BFRP-YD-J		(25Y→50D) × 5		5
	BFRP-YD-K		(25Y→50D) × 5→25Y		5
	BFRP-YD-L		(25Y→50D) × 6		5

**Table 3:** Arrangement of SEM scanning specimens.

Corrosion Environment	Specimen Number	Corrosion Duration	Number
Control group	SEM-O	-	1
Chloride environment	SEM-S1	75 d	1
	SEM-S2	150 d	1
Freezing and thawing cycle	SEM-F1	150 times	1
	SEM-F2	300 times	1
Chloride freeze-thaw coupling environment	SEM-SF1	(25Y→50D) × 3	1
	SEM-SF2	(25Y→50D) × 6	1
Footing	-	-	7

**Table 4:** EDS energy spectrum scanning specimen arrangement.

Corrosion Environment	Specimen Number	Corrosion Duration	Number
Control group	EDS-O	-	1
Chloride environment	EDS-S	150 d	1
Freezing and thawing cycle	EDS-F	300 times	1
Chloride freeze-thaw coupling environment	EDS-SF	(25Y→50D) × 6	1
Footing	-	-	4

**Table 5:** Changes in the process of BFRP stretching under different environments.

Corrosion/Aging Environment	Failure Process		Destruction Form
	Loading	Near Destruction	
Control group	Makes 2–3 ‘bang bang‘ sounds	A ‘hissing’ sound accompanied by white crack propagation and subsequent damage; a small amount of loose silk ‘thrown out’	Burst type
Chloride	No sound was made	The time gap from ‘hoarseness’ sound to specimen failure becomes shorter and accompanied by slow expansion of white cracks; a small amount of loose silk ‘thrown out’	The degree of cracking is small
Freezing and thawing cycle	Sends out 0–1 ‘bang bang‘ sound	The ‘hoarseness’ sound of the specimen failure time gap becomes shorter and is accompanied by the rapid expansion of white cracks; a large number of loose filaments ‘thrown out’	The degree of burst is large
Chloride freeze-thaw cycle	No sound was made	The time gap from ‘hoarseness’ sound to specimen failure becomes shorter and is accompanied by the slow expansion of white cracks; a large number of loose filaments ‘thrown out’	The degree of cracking is small

**Table 6:** The specific value of tensile strength decrease in BFRP sheets under different environmental conditions.

Chloride Environment			Freezing and Thawing Cycle			Chloride Freeze-Thaw Environment Coupling		
Specimen number	Tensile strength (MPa)	Percentage decrease (%)	Specimen number	Tensile strength (MPa)	Percentage decrease (%)	Specimen number	Tensile strength (MPa)	Percentage decrease (%)
D0	815.55	-	D0	815.55	-	D0	815.55	-
Y-A	808.53	0.86	D-A	812.31	0.40	YD-A	799.66	1.95
Y-B	798.72	2.06	D-B	796.71	2.31	YD-C	753.87	7.56
Y-C	788.56	3.31	D-C	781.01	4.23	YD-E	730.39	10.44
Y-D	766.66	5.99	D-D	774.34	5.05	YD-G	707.22	13.28
Y-E	746.59	8.46	D-E	752.71	7.71	YD-J	640.38	21.48
Y-F	737.06	9.62	D-F	722.05	11.16	YD-L	605.72	25.73

**Table 7:** The specific value of elastic modulus decrease in BFRP sheets under different environmental conditions.

Chloride Environment			Freezing and Thawing Cycle			Chloride Freeze-Thaw Environment Coupling		
Specimen number	Elastic modulus (MPa)	Percentage decrease (%)	Specimen number	Elastic modulus (MPa)	Percentage decrease (%)	Specimen number	Elastic modulus (MPa)	Percentage decrease (%)
D0	31.08	-	D0	31.08	-	D0	31.08	-
Y-A	30.85	7.4	D-A	31.14	−0.19	YD-A	30.99	0.29
Y-B	30.41	2.16	D-B	31.17	−0.29	YD-C	30.80	0.90
Y-C	30.20	2.83	D-C	30.46	1.99	YD-E	30.12	3.09
Y-D	30.15	2.99	D-D	30.27	2.61	YD-G	28.76	7.46
Y-E	29.44	5.28	D-E	30.15	2.99	YD-J	28.22	9.20
Y-F	28.90	7.01	D-F	28.95	6.85	YD-L	28.11	9.56

**Table 8:** The specific value of the decrease in the elongation at break of BFRP sheets under different environmental effects.

Chloride Environment			Freezing and Thawing Cycle			Chloride Freeze-Thaw Environment Coupling		
Specimen number	Breaking elongation (%)	Percentage decrease (%)	Specimen number	Breaking elongation (%)	Percentage decrease (%)	Specimen number	Breaking elongation (%)	Percentage decrease (%)
D0	3.38	-	D0	3.38	-	D0	3.38	-
Y-A	3.36	0.59	D-A	3.31	2.07	YD-A	3.30	2.37
Y-B	3.28	2.96	D-B	3.28	2.96	YD-C	3.18	5.92
Y-C	3.27	3.25	D-C	3.23	4.44	YD-E	3.06	9.47
Y-D	3.24	4.14	D-D	3.13	7.40	YD-G	2.90	14.20
Y-E	3.13	7.40	D-E	3.06	9.47	YD-J	2.63	22.19
Y-F	3.03	10.36	D-F	2.94	13.02	YD-L	2.54	24.85

## Data Availability

The data presented in this study are available on request from the corresponding author.

## Abbreviations

The following abbreviations are used in this manuscript:

BFRP	Basalt fiber reinforced polymer
25Y	25 days of chloride salt immersion
50D	50 freeze-thaw cycles, and ‘*’ is the number of couplings
